# Recurrent furunculosis as a cause of isolated penile lymphedema: a case report

**DOI:** 10.1186/1752-1947-4-196

**Published:** 2010-06-29

**Authors:** Ali A Al-shaham, Suneet Sood

**Affiliations:** 1 Faculty of Medicine, Universiti Teknologi MARA, Level 11, Dean's Office, Selayang Hospital, Selayang-Kepong Road, Batu Caves, Selangor 68000, Malaysia

## Abstract

**Introduction:**

Isolated lymphedema of the penis is extremely rare: combined involvement of the scrotum and penis is the norm. Furunculosis as a cause is not, to our knowledge, previously reported. We present a case of isolated penile lymphedema that responded to excision of lymphedematous tissue and reconstruction with flaps.

**Case presentation:**

A 32-year-old Arab man presented with a three-year history of a gradually increasing, painless penile swelling. Our patient's main complaint was non-erectile sexual dysfunction. The swelling was preceded by at least three prior episodes of severe furunculosis at the penile root. He had no other contributory past medical or family history. On examination there was gross penile enlargement, maximally at the mid shaft, associated with thickened skin at the sites of prior furunculosis. The glans and scrotum were normal. Both testes were palpable. Serology for filariasis, and urinary tract ultrasound and computed tomography scan were normal. The clinical diagnosis was lymphedema following recurrent penile furunculosis. At operation the lymphedematous tissues were removed. Closure of the penile shaft was accomplished by bilateral advancement of flaps from both ends of the penis. He resumed normal sexual activity one month after surgery. At 12 months, he had a good cosmetic result, with no signs of recurrence.

**Conclusions:**

Furunculosis at the penile root may result in lymphedema confined to the penile shaft, sparing the scrotum. Excision of abnormal tissue and cover with a skin flap gave excellent cosmetic results, and allowed satisfactory sexual activity.

## Introduction

Although peno-scrotal lymphedema is common, isolated penile lymphedema is extremely rare [1]. A literature review for the last 30 years revealed only two cases of isolated penile involvement. In the first, chronic lymphatic edema occurred due to long term usage of a penile ring [2]. In the second case [3] the penile swelling resulted from donovanosis. We present a case of isolated penile lymphedema that was caused by recurrent skin infection.

Genital lymphedema is an accumulation of lymph in the superficial lymphatic channels between skin and fasciae: Colles' fascia in the scrotum and Buck's fascia in the penis [4]. The testes, the spermatic cord, the corpora, and the glans drain into the deep lymphatics, and are not affected by the disease process. Penoscrotal lymphedema is disfiguring. It is also associated with pain, difficulties with urination, and sexual dysfunction [5,6]. Diagnosis of lymphedema is easy, but determining its etiology is much more difficult. Conditions that damage the penile lymphatics, resulting in lymphedema, include neoplasia, surgical trauma and radiation. Chronic or recurrent acute infection may also cause lymphedema. Examples include donovanosis, tuberculosis and parasitic diseases such as filariasis [7]. Primary lymphedema is less common, and is due to an intrinsic abnormality in lymphatic channels, such as lymphatic aplasia, hypoplasia and hyperplasia. Elephantiasis is a term usually applied to long standing lymphedema with numerous folds and variable thickening in the skin and hardening of the subcutaneous tissue due to fibrosis.

## Case presentation

A 32-year-old Arab man presented with a three-year history of a painless fusiform swelling in the penis. The swelling was preceded by three episodes of furunculosis over a nine-month period, soon after which he developed this swelling. The infection itself was characterized by relapses and remissions, with the total duration of infection being four months within these nine months. The infection was associated with pus that discharged spontaneously through minute sinuses. It responded very slowly to systemic and local antibiotic treatment as well as improved personal hygiene. Eventually healing was accomplished, leaving the penile skin thickened and irregular.

**Figure 1:**
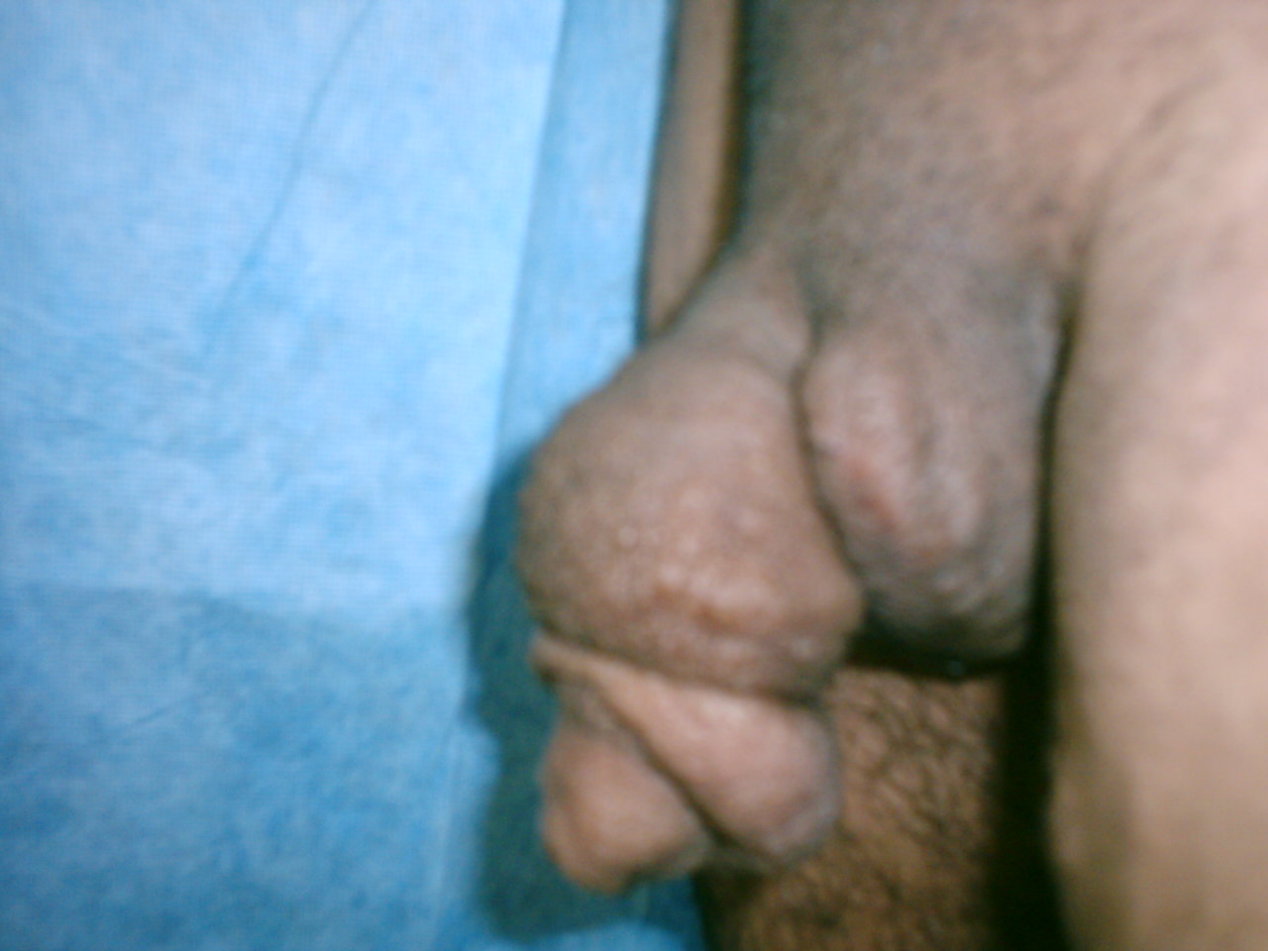
**Pre-operative views of the patient with penile lymphedema**.

The onset of the swelling was gradual, with edema that spread circumferentially and peripherally along the penile shaft. Our patient's main complaint was non-erectile sexual dysfunction. He had no urinary tract symptoms. There was no history of irradiation, surgery, trauma, or travel to areas endemic with filariasis. He denied a contributory family history. On examination he had gross penile enlargement, maximally at the mid shaft, where the circumference was 21 cm. The skin was thick, with numerous irregular shallow folds (Figure 1). The glans penis, penile root, scrotal sac, thigh and perineal skin were normal. Both testes could be identified easily. He maintained poor local hygiene. Serology for filariasis was normal, and ultrasound of testes and lower urinary tract as well as abdominal computed tomography (CT) scan showed no abnormality. The clinical diagnosis was secondary lymphedema following recurrent penile furunculosis.

**Figure 2:**
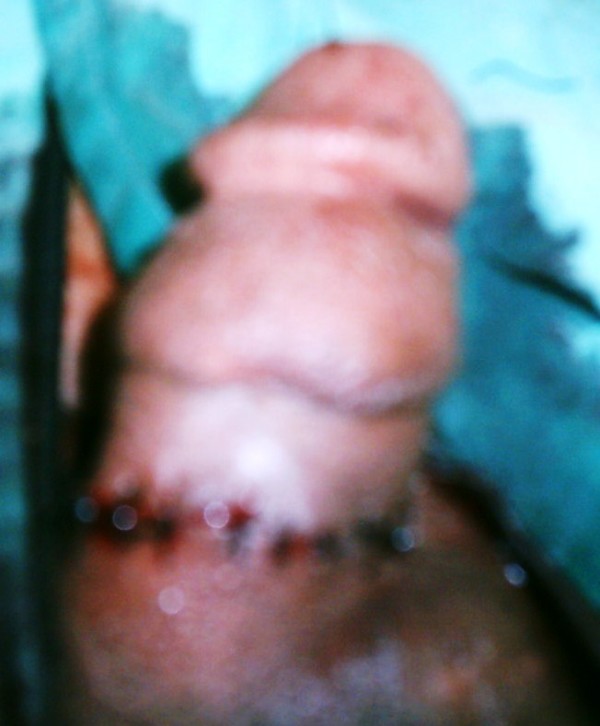
**Bilateral advancement of sleeve-like flaps to ensure closure**.

We planned surgery for our patient. Written informed consent was obtained after an explanation of the procedure and expected outcomes. The operation was started by two circular incisions around the penile shaft at the penile corona and root level, followed by a median dorsal incision made through skin and subcutaneous tissue. The incisions were carefully extended down to Buck's fascia from the penile root to the coronal sulcus. The two lateral lymphedematous segments of tissue were separated from the fascia by sharp dissection, leaving the penile shaft exposed. Closure of the penile shaft was accomplished by bilateral advancement of sleeve-like flaps from both ends of the penis and suture with monofilament non-resorbable suture (Figure 2).

Histopathologic examination showed non-specific chronic inflammation with areas of epidermal thickening and dermal fibrosis.

The wound healed well, and sutures were removed on the 14th post-operative day. Antibiotic cover with penicillin was administered one day pre-operatively and for 14 days post-operatively. He made an uneventful recovery. He had residual edema in the reflected mucosa of the coronal sulcus, which disappeared within one month. He resumed a normal sexual activity one month after surgery. At 12 months, he had a good cosmetic result, with no signs of recurrence.

## Discussion

Male genital lymphedema typically involves the scrotum, and involvement of the penile shaft occurs late. Isolate penile lymphedema may result from surgery, irradiation, or from certain chronic infections.

Long standing penile skin infection with subsequent fibrosis in the subcutaneous tissue outside the tunica may obstruct the lymphatic channels leading to development of penile lymphedema. Genital lymphadema following recurrent furunculosis has not been reported. Our patient probably developed recurrent furunculosis due to poor personal hygiene, which was also evident at the time of the current presentation. In our patient the chronic infection caused extensive lymphatic damage at the penile root, with consequent lymphedema.

Although significant improvement is possible by a variety of methods, the treatment of choice in genital lymphedema is excision of all abnormal tissue [6-8]. This is easy; the challenge is in providing a cover to exposed areas. There are two options: a local skin flap and a split skin graft [7-11]. The posterior scrotal and the perineal skin have a collateral lymphatic drainage and are usually available and can be used to generate flaps for scrotal reconstruction.

Dandapat *et al*. [6], reviewing 350 cases, concluded that satisfactory results were achieved by using local flaps to the scrotum. They recommended split thickness skin grafts for penile cover. Tapper *et al*. [12] were able to cover the scrotum and penis by local skin flaps after excision of the lymphedematous tissue. The results were satisfactory at one year of follow up. Longer follow up was required in predicting the recurrence, especially in flap cases. Yormuk *et al*. [8] described a case where surgical excision was performed for recurrent lymphedema. They used a local flap to cover the scrotum and a split thickness skin graft for the penile cover. There was a good result at five years of follow up.

The unavailability of nearby normal skin to cover the penile part by flaps leaves surgeons with only the choice of skin graft. McDougal [13] suggested that the inner preputial skin can be spared and used for partial penile coverage in uncircumcised patients. Alternatively, sleeve-like flaps at both ends, when available, can be advanced bilaterally to provide good cover, as in our case. The flap has better cosmetic results, but a higher incidence of local recurrence [6,13]. Malloy *et al*. [5] advocated the use of a split skin graft for penoscrotal elephantiasis after they noticed some cases of recurrence in the local flap. The split skin graft has the advantage of lower recurrence rates, but the cosmetic results are poorer [4,6,8,9] and the subsequent contraction in the skin graft decreases the graft elasticity: this imposes additional problems during erection. We suggest that a flap provides penile cover with better cosmesis, low contraction rates, and adequate elasticity for erection. If one is compelled to use a graft, a full thickness graft (Wolfe) may be a better choice than a split thickness graft. Mesh grafts [11] yield poor cosmetic results, and are rarely required.

## Conclusions

Long standing skin infection with subsequent fibrosis may lead to development of penile lymphedema. We present a patient who developed isolated penile lymphedema following recurrent furunculosis. After lymphedema excision, cover of the denuded penile shaft by tailored skin flaps provided excellent cosmetic results and good sexual function.

## Consent

Written informed consent was obtained from the patient for publication of this case report and any accompanying images. A copy of the written consent is available for review by the Editor-in-Chief of this journal.

## Competing interests

The authors declare that they have no competing interests.

## Authors' contributions

AA saw and operated on the patient. SS was the major contributor in the preparation and revision of the manuscript. Both authors have read and approved the final version of the manuscript.
